# 

**Published:** 1897-03

**Authors:** 


					﻿CONTENTS.
ORIGINAL ARTICLES—
Treatment of United Fractures. By William Perrin Nicolson,
M. D., Atlanta, Ga...................................  101
Acute Lobar Pneumonia—Treatment Specially. By Ramon D.
Garcin, M. D., Richmond, Va........................   104
Tubercular Meningitis. By J. Allison Hodges, M. D., Richmond,
Va.................................................... 107
SELECTIONS AND ABSTRACTS—
Puerperal Eclampsia; Its Etiology and Treatment........ 112
Our Immunity from Plague............................... 113
Coitus as a Disturbing Element........................  116
Calcium Sulphydrate; A Safe, Efficient and Economical Depila-
tory.................................................  118
Some Observations on Medical Topics in Fiction......... 120
Sea Water and Menstruation............................. 123
Diagnosis of Carcinoma of the Breast in its Early Stages. 123
Interaction of Somatic and Psychic Disorders.'...... 125
Albumin in the Urine.............................    128
The Associated Study of Crime and Insanity.......... 132
Venereal Buboes...................................   133
Pregnancy Diagnosed by the Urine..................   133
The Medical Treatment of Inebriety.................. 134
A Town Enslaved by the Cocaine Habit................ 135
Vaginal Ligation of the Uterine Arteries for Uterine Fibromata, 136
The Ice-Bag in Pneumonia............................ 136
Diabetes in a Young Child..........................  137
Etiology of Acute Rheumatism.......................  138
A Russian Horror...................................  139
EDITORIAL.............................................  140
CORRESPONDENCE...................................    ...	142
BOOK REVIEWS........................................... 143
SPECIAL NOTES.......................................    145
				

